# Blastic Plasmacytoid Dendritic Cell Neoplasm: A Case Report of a Rare and Aggressive Hematologic Malignancy

**DOI:** 10.7759/cureus.71849

**Published:** 2024-10-19

**Authors:** Kaushik Saha, Gowthami Sai Kumari Thoram, Soumyajit Roychoudhury

**Affiliations:** 1 Department of Pathology, Murshidabad Medical College and Hospital, Berhampore, IND; 2 Dermatology, Rejuven Skin Clinic, Berhampore, IND

**Keywords:** blastic plasmacytoid dendritic cell neoplasm (bpdcn), cd4+cd56+, nk cell, plasmacytoid dendritic cell (pdc), skin lymphoma, tagraxofusp

## Abstract

Blastic plasmacytoid dendritic cell neoplasm (BPDCN) is a strikingly unusual, clinically challenging, and rapidly spreading tumor that originates from plasmacytoid dendritic cell (PDC) precursors. It has a high incidence of skin and bone marrow involvement as well as leukemic dissemination. It shows a considerable biologic diversity with overlapping morphologic and immunophenotypic features of various cutaneous hematolymphoid neoplasms. Studies with large series of patients are not available due to low prevalence and short survival of the disease. We report here a rare case of BPDCN in a 30-year-old male patient who primarily came with skin manifestations almost all over the body surface. Skin biopsy revealed monomorphic medium-sized undifferentiated blast-like cells filling the entire dermis sparing the epidermis. The cells were immunopositive for CD45, CD56, CD4, and CD123 with a high Ki-67 labeling index while they were negative for known B-cell and T-cell markers. Radiological evaluation revealed lymphadenopathy at various sites. Peripheral blood smears and bone marrow aspiration smears demonstrated similar blast-like cells. Misdiagnosis or late diagnosis of this clinically heterogeneous BPDCN may lead to systemic spread and poor outcomes. Hence, prompt diagnosis and treatment are essential, with a multidisciplinary approach.

## Introduction

Blastic plasmacytoid dendritic cell neoplasm (BPDCN) is a rare clinically aggressive tumor without any racial or ethnic predilection. It derives from the precursors of PDCs. It has a high incidence of cutaneous and bone marrow involvement as well as leukemic dissemination. It was previously known as blastic NK-cell lymphoma or agranular CD4+CD56+ hematodermic tumor [1]. It is much more common in elderly people, but it has also been reported in other age groups, including children. It is three times more common in males than in females [1,2]. BPDCN is responsible for less than 1% of acute leukemias and cutaneous lymphomas [3]. BPDCNs are not only clinically heterogeneous but they also show a considerable biologic diversity with overlapping morphologic and immunophenotypic features of various cutaneous hematolymphoid neoplasms [4,5]. In addition, the low prevalence and short survival of the disease have hampered the study of large series of patients [5]. We report here a case of BPDCN in a 30-year-old male who primarily turned up with skin lesions.

## Case presentation

**Figure 1:**
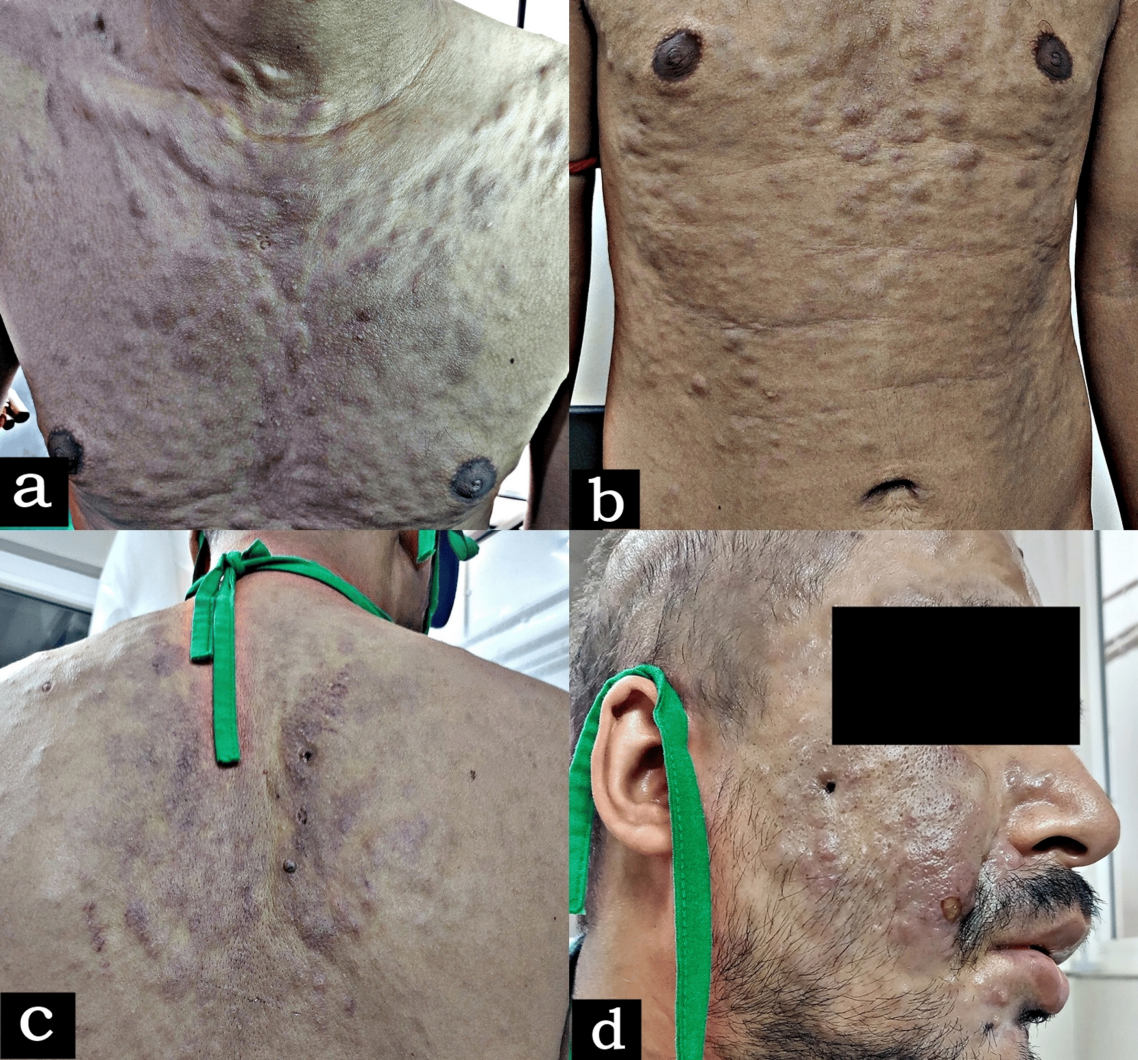
Multiple erythematous-to-violaceous macules, nodules, and plaques over the (a) anterior chest wall, (b) anterior abdominal wall, (c) back, and (d) face and scalp

A 30-year-old male patient presented with multiple erythematous-to-violaceous macules, nodules, and plaques almost all over the body surface (Figure 1).

**Figure 2:**
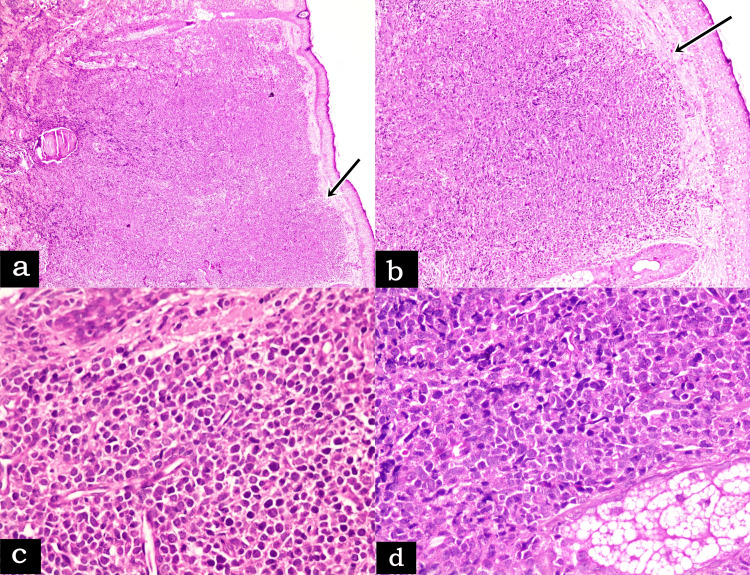
(a) Photomicrograph showing scanner view (H&E, ×40), and (b) Low power view (H&E, ×100) of heavy dermal infiltrate with free epidermis and uninvolved Grenz zone (marked by arrow) in skin biopsy; photomicrograph showing high-power view (c) and (d) (H&E, ×400) of medium-sized blast-like undifferentiated cells with scanty agranular cytoplasm and one or two prominent nucleoli H&E: hematoxylin and eosin

The consultant dermatologist immediately arranged for a skin biopsy from multiple sites, suspecting it as a case of skin lymphoma. Skin biopsy sections on routine hematoxylin and eosin (H&E) staining revealed diffuse and massive involvement of the entire dermis with extension to the subcutaneous fat by atypical cells sparing the epidermis with an uninvolved Grenz zone underneath (Figure 2). The neoplastic cells were monomorphic, medium-sized, undifferentiated, blast-like cells having irregular, eccentrically located nuclei, finely dispersed nuclear chromatin, one to two small but conspicuous nucleoli, scanty agranular cytoplasm, and 3-5 mitotic figures/high-power field (HPF).

**Figure 3:**
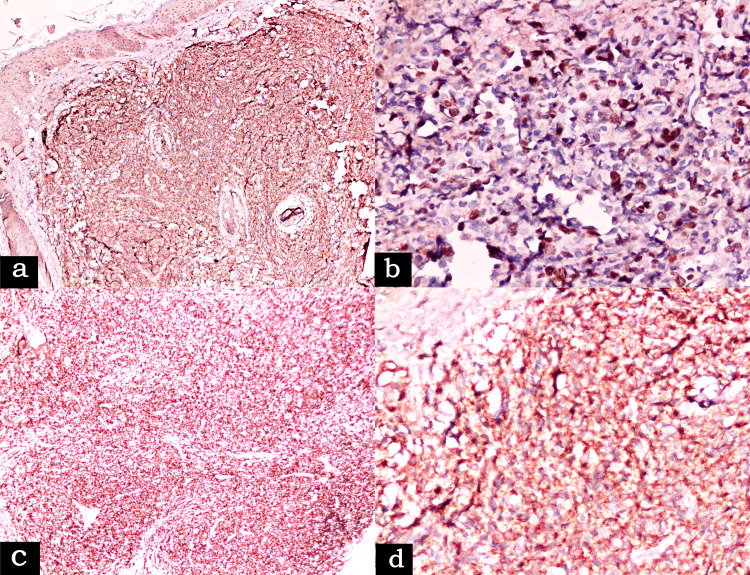
(a) Positive immunostaining of malignant cells with CD56 (×100), (b) high Ki67 labeling index (×400), (c) positive expression of CD4 (×100), and (d) CD123 (×400)

By immunohistochemistry (IHC), the cells were immunopositive (Figure 3) for CD45, CD56, CD4, CD123, CD7 (patchy), TdT (patchy) while negative for CD3, CD5, CD20, CD79a, CD138, MPO, MUM1, PAX5, CD33, CD30, CD34, S100, Alk, chromogranin, synaptophysin, and EMA. The Ki67 labeling index (LI) was 58%.

**Figure 4:**
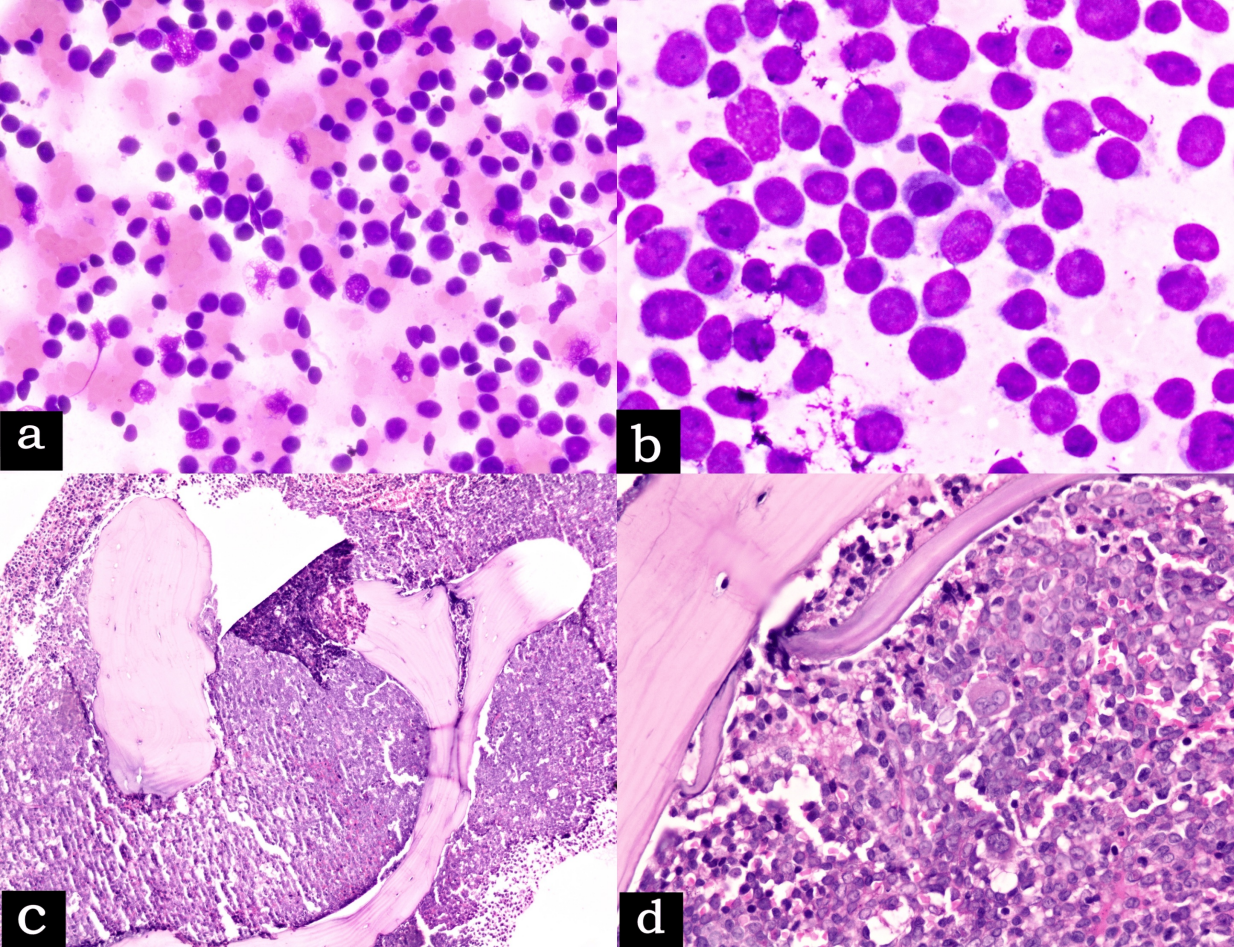
(a) Bone marrow aspiration cytology (MGG, ×400), (b) bone marrow imprint smear (MGG, ×1000) showing monomorphic undifferentiated lymphoblast-like cells, (c) low-power view (H&E, ×100), and (d) high-power view (H&E, ×400) of bone marrow biopsy sections demonstrated diffuse infiltration of undifferentiated blast-like cells. H&E: hematoxylin and eosin

Pancytopenia, relative lymphocytosis, and 25% blast-like cells were noted in the complete blood count. Radiological evaluation revealed bilateral cervical, axillary, mediastinal, iliac, retroperitoneal, and inguinal lymph nodes. The liver and spleen were not palpable on physical examination and unremarkable on abdominal ultrasound as well. Peripheral blood smear, bone marrow aspiration smear (Figure 4a), and bone marrow imprint smear (Figure 4b) demonstrated monomorphic, undifferentiated, lymphoblast-like cells with a high N: C (nucleocytoplasmic) ratio, one to two prominent nucleoli, fine nuclear chromatin, and a moderate amount of bluish agranular cytoplasm without any evidence of Auer rods. Greater than 70% involvement of bone marrow biopsy sections (Figure 4c and 4d) with diffuse infiltrate of the neoplastic cells. Subsequent lymph node biopsy also displayed diffuse involvement of the medulla and interfollicular areas with few residual follicles. Liver and kidney functions and coagulation profile were normal while routine viral markers were negative. Lactate dehydrogenase (LDH) and erythrocyte sedimentation rate (ESR) were moderately high. The patient was referred to a higher center for further management. That is why no other data is available on the therapy and survival of the patient.

## Discussion

The term BPDCN was first described in the fourth edition of the World Health Organisation (WHO) classification in 2008 [6], and it was categorized under acute myeloid leukemia (AML) and related precursor neoplasms. The revised fourth edition of the WHO classification in 2017 mentioned BPDCN as a separate distinct entity [1]. In the most recent WHO fifth edition, BPDCN is classified under dendritic cell and histiocytic neoplasms. Mesenchymal tumors specific to lymph nodes and spleen are separately classified as stroma-derived neoplasms of lymphoid tissues [7,8]. The true incidence of BPDCN is unrevealed, partly due to its various names and partly due to the rarity of the disease. The prognosis of BPDCN is extremely poor, with a median overall survival of around one year. Varying presentations and a diverse array of cutaneous lesions create a huge challenge for a clinician. A fast and definitive diagnosis needs relevant clinical knowledge and conclusive opinion by a senior pathologist and the use of a wide panel of IHC markers [9]. BPDCN needs to be differentiated from mature plasmacytoid dendritic cell proliferation (MPDCP), AML/myeloid sarcoma, B-cell or T-cell lymphoblastic leukemia/lymphoma (B-ALL/LBL or T-ALL/LBL), and neuroendocrine tumor on morphological basis. A panel of IHC markers can weed out most of the differentials. Low-grade morphology, scanty to absent mitosis, association with myeloid neoplasm, very low Ki67 index, and negative expression of CD56 indicate MPDCP. In AML/myeloid sarcoma, MPO, CD117, CD33, CD34, and TdT are positively expressed but B- and T-lineage markers are negative. IHC markers like CD20, CD79a, CD19, PAX5, and TdT are positive in B-ALL/LBL, and CD3, CD5, CD4, CD8, and TdT are positive in T-ALL/LBL. B-ALL/LBL and T-ALL/LBL both are negative for myeloid lineage markers like MPO, CD117, CD33, etc. [1,10]. Treatment options for this aggressive disease range from conventional polychemotherapy to the recently approved CD123-targeted fusion protein tagraxofusp. However, stem cell transplant (SCT) is the only potentially curative therapy for BPDCN [11,12].

## Conclusions

The growing knowledge and development of definite diagnostic criteria over the last few years helped us to design a robust diagnostic workup protocol with a wide panel of IHC markers. Similarly, in our case, we arrived at the final diagnosis after performing a long list of IHC markers considering BPDCN from histomorphology and clinical history. However, there is still a high risk of misdiagnosis or delay in the correct diagnosis leading to disease spread, progression to systemic involvement, and poor survival. Hence, it is essential to think about BPDCN in relevant cases, arrange a skin biopsy rapidly for confirmation, and start therapy immediately.
